# Editorial: Ecological and Evolutionary Aspects of Complex Relations Between Micro- and Macroparasites and Their Wild Animal Hosts

**DOI:** 10.3389/fvets.2019.00496

**Published:** 2020-01-17

**Authors:** Serge Morand, Michael Kosoy

**Affiliations:** ^1^Centre National de la Recherche Scientifique, Centre de Coopération Internationale en Recherche Agronomique pour le Développement, Montpellier University, Paris, France; ^2^Faculty of Veterinary Technology, Kasetsart University, Bangkok, Thailand; ^3^KB One Health LLC, Fort Collins, CO, United States

**Keywords:** one health, wildlife, zoonotic disease, evolutionary ecology, disease ecology, conservation medicine

Disease ecology emphasizes how ecological interactions between microparasites (pathogenic microbes), macroparasites (helminths, protists), and animal hosts help understanding transmission of diseases and parasites within the epidemiological landscape. Diseases and parasites are ecological and evolutionary forces at all biological levels of organizations from organisms, populations, communities to ecosystems and there is an increasing interest to investigate them in an evolutionary ecological perspective.

Integrating evolution, co-evolution into the ecology of transmission in a spatial context poses many challenges. To tackle these challenges disease ecologists use a wide varieties of methods and tools such as molecular approaches developed from pathogens screening, high-throughput technologies, population genetics, phylogenetics and phylogeography, quantitative epidemiology, population dynamics, theoretical epidemiology, spatial analyses, and landscape ecology.

This special issue calls into presenting advances and identifying gaps in the disease ecology and evolutionary ecology of diseases, using wild mammals and their pathogenic bacteria, viruses, parasites, and vectors as so many models. This special issue is a collection of studies in disease ecology that contribute to Conservation Medicine (1) and One Health approaches (2).

In two studies presented in this special issue, bats and their parasites, vectors, and microbes were investigated. Information on bats, bat flies (obligate hematophagous ectoparasites of bats) and their microparasites was synthesized by Szentiványi et al. Viruses, bacteria, blood protists, and fungi have been detected in bat flies that show physiological consequences on bats and their ectoparasites. The authors recommended additional studies to understand the interlinkages between bat hosts, ectoparasites, and their associated microparasites. McKee et al. examined *Bartonella* from European bats and their ectoparasites using network analysis, Bayesian phylogenetics, and tests on co-phylogenetic association. The authors were able to disentangle the processes, ecological, or evolutionary, that contribute to shape the interactive communities. Bat phylogeny and bat roost sharing help to explain the evolutionary patterns of vector-borne diseases.

Carnivores and bacterial diseases were the topic of two studies. Kosoy and Goodrich reviewed published studies on the phylogenetic sister clades *Bartonella* and *Brucella* that infect wild carnivores to analyse and compare the ecology of these two clades of bacteria in closely related host species. *Bartonella* species were much reported in every sampled wild felid species, whereas among *Brucella* studies only few of them have reported *Brucella* in felids by detection of antibodies. The authors stressed that wild carnivores often carry the same microparasites as the domesticated cats and dogs, merely exposure is related to differences in biology, distribution, and historical interactions. In a comprehensive review, André synthesized the actual knowledge on the diversity of the tick-borne bacteria of species from *Ehrlichia, Anaplasma* and “*Candidatus Neoehrlichia* sp.” in wild carnivores worldwide and discussed consequences for human and animal health as well as wildlife conservation. The author emphasized the importance of Whole Genome Sequencing and Next Generation Sequencing (NGS) technologies to better understand the importance of wild carnivores in the transmission of several agents such as Anaplasmataceae.

Flea-borne rickettsial disease was further explored by Maina et al. who summarized and discussed the actual knowledge of the epidemiology and distribution of *Rickettsia asembonensis*, a well-characterized rickettsia of the *Rickettsia felis*-like organisms, worldwide, as well as its arthropod hosts. The authors emphasized the need to conduct further analyses, functional and structural, to find out differences and/or similarities between *R. asembonensis* and other rickettsial species, and to better characterize the current/potential arthropod vectors with other flea-borne rickettsial species (*R. felis* and *R. typhi)*, but also non-rickettsial pathogens such as *Yersinia pestis*, the agent of the plague.

Sariyeva et al. investigated the role of gray marmots in the maintenance of highly virulent strains of *Y. pestis* in endemic foci of the Tien Shan Mountains, Kyrgyzstan. Plague circulates incessantly in populations of gray marmots, their fleas and other rodent species, stressing the importance of significant changes in rodent communities during the previous two decades. Biggins and Eads reviewed hypotheses regarding the epidemiology of *Y. pestis* using recent data from North America supporting maintenance of *Y. pestis* by persistent transmission. They proposed a maintenance mechanism, the Synergistic Positive Feedback cycles, that facilitates periodic epizootic eruptions “in place” resulting in sudden outbreaks that spread rapidly in time and space involving flea vectors, hosts, and the plague bacterium. The authors stressed that the absence of plague epizootics may reduce public health risk, but may still have ecologic impact on wild mammalian populations.

Studies on the parasites and diseases find application in conservation medicine as exemplified by Tangtrongsup et al. who investigated the prevalence of intestinal parasites of *Giardia duodenalis* and *Cryptosporidium spp*. in captive agile gibbons (*Hylobates agilis*), lar gibbons (*H. lar*), and pileated gibbons (*H. pileatus*) at the Krabokkoo Wildlife Breeding Center, Thailand. The authors stressed the improvement of hygiene management to prevent potential transmission between gibbon and human.

Finally, two studies contributed to the One Health approach. Using a metagenomic approach, Takhampunya et al. conducted an intensive study in populations of humans, animals, and vectors in Northern Thailand where scrub typhus is highly endemic. *Leptospira* spp., *Bartonella* spp., *Rickettsia* spp., and *Orientia tsutsugamushi* were detected using NGS in the studied populations. The authors confirmed the transmission of several bacterial diseases in the area, some of which are known to cause severe illness in human populations. Ruiz-Arrondo et al. outlined the benefits and the limitation of the entomological surveillance programme of mosquitoes implemented by the Government of La Rioja (Northern Spain). In order to implement a One Health approach, the surveillance programme should screen wild birds for flaviviruses and sentinel horses. Better coordinating efforts from biologists, epidemiologists, and veterinarians would be an added value to enable ecological data to be operationalised to inform human, animal, and ecosystem health.

## Author Contributions

SM and MK have served as editors of the Research Topic and have co-written the editorial.

### Conflict of Interest

MK was employed by company KB One Health LLC. The remaining author declares that the research was conducted in the absence of any commercial or financial relationships that could be construed as a potential conflict of interest.
